# Supplementary material for: First Report and Characterization of a Plasmid-Encoded blaSFO-1 in a Multi-Drug-Resistant Aeromonas hydrophila Clinical Isolate
Source: Microorganisms. 2024 Feb 29;12(3):494. doi: 10.3390/microorganisms12030494 (PMC10974073; doi:10.3390/microorganisms12030494)
Supplement: Supplementary file 1 [file microorganisms-12-00494-s001.zip › Supplemental Figures and Tables.pdf]

Supplemental Figures and Tables

Supplemental Table S1. Genes used to perform phylogenetic analysis of MAH-4 with representative *Aeromonas* species

Product	PGFam	Align. Score	Align. Length	Num Seqs	Mean Sqr Freq	Prop Gaps
Glycine dehydrogenase [decarboxylating] (glycine cleavage system P protein) (EC 1.4.4.2)	PGF_00008773	29.08	958	26	0.94	0.007
DNA gyrase subunit B (EC 5.99.1.3)	PGF_06703483	27.57	804	26	0.972	0.001
BioD-like N-terminal domain / Phosphate acetyltransferase (EC 2.3.1.8)	PGF_01937476	25.61	727	26	0.95	0.02
Oligopeptidase A (EC 3.4.24.70)	PGF_00026904	23.9	680	26	0.917	0.011
Sensory box sensor/GGDEF/EAL domain protein	PGF_00050892	23.37	893	26	0.782	0.072
DNA primase DnaG	PGF_00421679	23.1	596	26	0.946	0.002
Phosphoenolpyruvate carboxykinase [ATP] (EC 4.1.1.49)	PGF_02947204	22.28	546	26	0.954	0.009
NAD synthetase (EC 6.3.1.5) / Glutamine amidotransferase chain of NAD synthetase	PGF_06525160	22.05	540	26	0.949	0
Alkyl hydroperoxide reductase protein F	PGF_09969389	22.04	530	26	0.958	0.006
Hydroxylamine reductase (EC 1.7.99.1)	PGF_00736089	21.88	550	26	0.933	0.002
2-isopropylmalate synthase (EC 2.3.3.13)	PGF_04294677	21.88	524	26	0.956	0.004
Guanosine-5'-triphosphate,3'-diphosphate pyrophosphatase (EC 3.6.1.40) @ Exopolyphosphatase (EC 3.6.1.11)	PGF_05837876	21.26	498	26	0.952	0.004
ATP synthase beta chain (EC 3.6.3.14)	PGF_05195027	21.1	462	26	0.982	0
Methylcrotonyl-CoA carboxylase carboxyl transferase subunit (EC 6.4.1.4)	PGF_05369673	20.91	549	26	0.892	0.025
IMP cyclohydrolase (EC 3.5.4.10) / Phosphoribosylaminoimidazolecarboxamide formyltransferase (EC 2.1.2.3)	PGF_00013509	20.85	535	26	0.901	0.033
Glutamate-1-semialdehyde 2,1-aminomutase (EC 5.4.3.8)	PGF_09019918	19.97	429	26	0.964	0.002
tRNA cytosine(34) acetyltransferase (EC 2.3.1.193)	PGF_01956528	19.95	736	26	0.735	0.065
O-acetylhomoserine sulfhydrylase (EC 2.5.1.49) @ O-succinylhomoserine sulfhydrylase (EC 2.5.1.48)	PGF_04792526	19.83	422	26	0.965	0
Paraquat-inducible protein B	PGF_12719092	19.81	549	26	0.845	0.022
ATP-dependent RNA helicase RhlB (EC 3.6.4.13)	PGF_02213711	19.75	429	26	0.954	0.011
Outer membrane stress sensor protease DegQ, serine protease	PGF_03779043	19.67	469	26	0.908	0.031
Aromatic amino acid transport protein AroP	PGF_00064863	19.64	460	26	0.916	0.007
Putative Na+/H+ antiporter	PGF_10543494	19.25	444	26	0.914	0.037
Adenylosuccinate synthetase (EC 6.3.4.4)	PGF_06935032	19.21	418	26	0.939	0.002
Membrane-bound lytic murein transglycosylase F (EC 4.2.2.n1)	PGF_00020190	19.12	508	26	0.848	0.028
Murein peptide ABC transporter, substrate-binding protein (requires DppBCDF)	PGF_00030901	18.93	553	26	0.805	0.056
Murein DD-endopeptidase MepM	PGF_00023403	18.71	448	26	0.884	0.013

Two-component system sensor histidine kinase	PGF_00052313	18.7	449	26	0.882	0.031
Sodium/glutamate symporter	PGF_06816941	18.58	440	26	0.886	0.029
Probable low-affinity inorganic phosphate transporter	PGF_00037171	18.56	421	26	0.905	0.026
GTP-binding and nucleic acid-binding protein YchF	PGF_00007012	18.46	363	26	0.969	0
Succinyl-CoA ligase [ADP-forming] beta chain (EC 6.2.1.5)	PGF_00054528	18.27	388	26	0.927	0.025
Lipoprotein releasing system transmembrane protein LolC	PGF_08074580	17.93	439	26	0.856	0.07
Integral membrane protein	PGF_00557891	17.91	393	26	0.903	0.016
Probable Na+/H+-dicarboxylate symporter	PGF_00552628	17.88	399	26	0.895	0.026
6-phosphofructokinase (EC 2.7.1.11)	PGF_07421229	17.45	323	26	0.971	0
Biotin synthase (EC 2.8.1.6)	PGF_01400330	17.33	392	26	0.875	0.075
Glycyl-tRNA synthetase alpha chain (EC 6.1.1.14)	PGF_00009967	17.3	310	26	0.983	0.009
Porphobilinogen synthase (EC 4.2.1.24)	PGF_00489714	17.3	339	26	0.939	0.001
Transcriptional factor MdcH	PGF_00559497	17.22	333	26	0.944	0.014
Cytochrome c heme lyase subunit CcmH	PGF_06068352	17.16	427	26	0.831	0.012
Deoxyguanosinetriphosphate triphosphohydrolase (EC 3.1.5.1), subgroup 1	PGF_10089955	17.15	458	26	0.801	0.046
Phosphate ABC transporter, substrate-binding protein PstS (TC 3.A.1.7.1)	PGF_07668761	17.07	324	26	0.948	0
Branched-chain amino acid ABC transporter, permease protein LivH (TC 3.A.1.4.1)	PGF_06868199	17.03	308	26	0.97	0
Cell-division-associated, ABC-transporter-like signaling protein FtsX	PGF_03701810	16.96	318	26	0.951	0.003
Flagellar protein FlgT	PGF_09781823	16.8	431	26	0.809	0.095
Transcriptional activator NhaR	PGF_00057513	16.58	310	26	0.942	0.014
Holliday junction ATP-dependent DNA helicase RuvB (EC 3.6.4.12)	PGF_05049118	16.58	337	26	0.903	0.031
Glycerol-3-phosphate dehydrogenase [NAD(P)+] (EC 1.1.1.94)	PGF_00008611	16.41	334	26	0.898	0.035
Tryptophanase (EC 4.1.99.1)	PGF_03720595	16.38	490	26	0.74	0.041
tRNA dimethylallyltransferase (EC 2.5.1.75)	PGF_04807486	16.24	309	26	0.924	0.002
(2E,6E)-farnesyl diphosphate synthase (EC 2.5.1.10)	PGF_06784545	16.06	296	26	0.934	0
Formyltetrahydrofolate deformylase (EC 3.5.1.10)	PGF_00006320	15.94	283	26	0.948	0.015
33 kDa chaperonin HslO	PGF_10462808	15.88	295	26	0.925	0.003
Lipoprotein NlpI	PGF_00017407	15.76	319	26	0.883	0.037
Cardiolipin synthase (EC 2.7.8.-) phosphatidylethanolamine-utilizing, bacterial type ClsC	PGF_08028591	15.67	437	26	0.75	0.044
tRNA (guanine(37)-N(1))-methyltransferase (EC 2.1.1.228)	PGF_00413208	15.57	249	26	0.987	0.001
Uncharacterized efflux ABC transporter, ATP-binding protein YadG	PGF_06025565	15.47	334	26	0.847	0.09
Outer membrane porin OmpC	PGF_00027880	15.42	412	26	0.76	0.137

Phospholipid ABC transporter permease protein MlaE	PGF_07114837	15.38	259	26	0.956	0.004
FIG008480: zinc-binding hydrolase	PGF_02211096	15.27	265	26	0.938	0
Uridylate kinase (EC 2.7.4.22)	PGF_02923127	15.15	244	26	0.97	0.002
Rod shape-determining protein MreC	PGF_10369954	15.09	312	26	0.854	0.069
Bis(5'-nucleosyl)-tetraphosphatase, symmetrical (EC 3.6.1.41)	PGF_07763915	15.08	275	26	0.909	0.007
Cytochrome c-type biogenesis protein CcmC, putative heme lyase for CcmE	PGF_00420482	14.99	246	26	0.956	0
tRNA uridine 5-oxyacetic acid(34) methyltransferase (EC 2.1.1.-)	PGF_00049344	14.93	271	26	0.907	0.04
Formamidopyrimidine-DNA glycosylase (EC 3.2.2.23)	PGF_05740384	14.91	270	26	0.907	0.023
Tol-Pal system protein TolQ	PGF_00648054	14.75	229	26	0.975	0.001
Arginine ABC transporter, substrate-binding protein ArtI	PGF_00502921	14.71	246	26	0.938	0.004
Ribose ABC transporter, substrate-binding protein RbsB (TC 3.A.1.2.1)	PGF_12669939	14.64	356	26	0.776	0.181
Pyrroline-5-carboxylate reductase (EC 1.5.1.2)	PGF_07609122	14.62	276	26	0.88	0.007
Peptide ABC transporter, ATP-binding protein SapF	PGF_08049323	14.59	279	26	0.874	0.064
Histidine ABC transporter, permease protein HisM (TC 3.A.1.3.1)	PGF_00974916	14.43	230	26	0.952	0
hypothetical protein	PGF_00195010	14.43	287	26	0.852	0.007
NADPH-dependent 7-cyano-7-deazaguanine reductase (EC 1.7.1.13)	PGF_00024855	14.39	302	26	0.828	0.061
Zn-dependent protease with chaperone function PA4632	PGF_01922821	14.26	277	26	0.857	0.022
Ribonuclease III (EC 3.1.26.3)	PGF_03790040	14.25	224	26	0.952	0.005
Uncharacterized UPF0721 integral membrane protein	PGF_08139483	14.18	262	26	0.876	0.019
Stringent starvation protein A	PGF_00054301	14.13	209	26	0.977	0
23S rRNA (adenine(1618)-N(6))-methyltransferase (EC 2.1.1.181)	PGF_00415130	13.98	338	26	0.761	0.067
Oxidoreductase, Gfo/Idh/MocA family	PGF_00738912	13.91	313	26	0.786	0.037
Arginyl-tRNA--protein transferase (EC 2.3.2.8)	PGF_02992100	13.88	238	26	0.9	0.005
FIG000875: Thioredoxin domain-containing protein EC-YbbN	PGF_03863088	13.72	282	26	0.817	0.024
NADH pyrophosphatase (EC 3.6.1.22), decaps 5'-NAD modified RNA	PGF_00024615	13.63	267	26	0.834	0.03
FIG00003370: Multicopper polyphenol oxidase	PGF_05501316	13.61	251	26	0.859	0.022
Inner membrane protein YohK	PGF_00014196	13.46	234	26	0.88	0.026
Predicted hydrolase/acyltransferase	PGF_03831039	13.43	292	26	0.786	0.021
Outer membrane beta-barrel assembly protein BamD	PGF_05872952	13.43	305	26	0.769	0.164
rRNA (guanine-N(1))-methyltransferase	PGF_00556937	13.37	292	26	0.782	0.086
Translation elongation factor P	PGF_03799365	13.33	188	26	0.972	0
Phospholipid ABC transporter shuttle protein MlaC	PGF_10659937	13.33	211	26	0.918	0.005

LSU ribosomal protein L5p (L11e)	PGF_00016443	13.26	179	26	0.991	0
Polyphosphate glucokinase (EC 2.7.1.63)	PGF_04211832	13.25	265	26	0.814	0.076
Outer membrane protein OmpK	PGF_00028291	13.09	294	26	0.764	0.057
Quercetin 2,3-dioxygenase (EC 1.13.11.24) => YhhW	PGF_02380724	13.08	228	26	0.867	0.025
UDP-2,3-diacylglucosamine diphosphatase (EC 3.6.1.54)	PGF_00063937	13.08	254	26	0.821	0.048
Outer membrane porin OmpC	PGF_00557072	12.91	419	26	0.631	0.16
hypothetical protein	PGF_00255967	12.7	264	26	0.782	0.044
Putative transmembrane protein	PGF_01769544	12.69	186	26	0.931	0.007

Supplemental Table S2. Nucleotide sequence similarity to *A. hydrophila* pMAH-4 using BLAST®

Description	max score	Total score	Query cover	E-value	% identity	Accession length	Acc. #
Aeromonas caviae strain AC1520 plasmid pAC1520, complete sequence	66550	4.54E+05	75%	0	95.88%	253471	CP120943.1
Klebsiella pneumoniae strain KP1814 plasmid pKP1814-1, complete sequence	48230	1.35E+05	20%	0	100.00%	299858	KX839207.1
Klebsiella variicola strain 4253 plasmid p4253-imp, complete sequence	48230	1.36E+05	19%	0	100.00%	334271	CP135069.1
Klebsiella variicola strain SHET-01 plasmid pNDM-IMP-1, complete sequence	48230	1.45E+05	20%	0	100.00%	347317	CP050681.1
Klebsiella pneumoniae strain WH11 plasmid pWH11, complete sequence	48230	1.03E+05	16%	0	100.00%	325030	ON882017.1
Enterobacter asburiae strain AR2284-yvys plasmid pAR2284_1, complete sequence	48230	1.62E+05	21%	0	100.00%	373545	CP083831.1
Klebsiella quasipneumoniae strain SWMUF35 plasmid pA, complete sequence	48230	1.35E+05	20%	0	100.00%	311723	CP068445.1
Klebsiella pneumoniae strain KP294 plasmid pIMP4-KP294, complete sequence	48224	1.53E+05	20%	0	99.99%	349403	CP083446.1
Klebsiella pneumoniae strain KP19-2581 plasmid pKP19-2581_367k_HI5_, complete sequence	45493	85100	9%	0	100.00%	367802	CP120875.1
Citrobacter freundii strain IDR1800045912-01-00 plasmid p1C157, complete sequence	39506	66744	9%	0	100.00%	156725	CP054295.1
Aeromonas caviae strain SCLZS52 chromosome, complete genome	39500	1.74E+05	21%	0	99.99%	4718963	CP091176.1
Klebsiella pneumoniae plasmid pQL5-NDM-KPC, complete sequence	36863	81155	7%	0	99.99%	214266	OR253888.1
Pseudomonas mandelii JR-1 plasmid, complete sequence	29288	40648	6%	0	99.99%	410512	CP005961.1
Aeromonas caviae DNA, complete genome, strain: WP3-S18-ESBL-02	28987	1.08E+05	14%	0	99.94%	4860108	AP022013.1
Klebsiella quasipneumoniae strain C3001 plasmid pC3001-2-NDM, complete sequence	28509	33544	5%	0	100.00%	270400	CP039792.1
Klebsiella quasipneumoniae strain A708 plasmid pA708-1, complete sequence	28507	81589	12%	0	100.00%	238703	CP026369.1
Klebsiella aerogenes strain AR_0161 plasmid unnamed, complete sequence	28507	1.07E+05	15%	0	100.00%	451422	CP028952.1
Klebsiella pneumoniae strain A708 plasmid pA708-IMP, complete sequence	28507	81589	12%	0	100.00%	238703	MF344567.1
Klebsiella quasipneumoniae subsp. quasipneumoniae plasmid p2019SCSN059_tmexCD_333k, complete sequence	28502	1.19E+05	15%	0	99.99%	333095	ON169978.1
Klebsiella pneumoniae strain KP21300 plasmid pIMP_KP21300, complete sequence	28498	85728	11%	0	99.98%	279261	CP124694.1
Klebsiella variicola plasmid pFK2020ZBJ35_tmexCD_325k, complete sequence	27835	55700	9%	0	99.99%	325393	ON169979.1
Klebsiella michiganensis strain 7525 plasmid pKOX7525_1, complete sequence	27215	1.33E+05	18%	0	99.99%	397447	CP065475.1
Aeromonas sp. ASNIH2 chromosome, complete genome	26328	86766	13%	0	99.99%	4801408	CP026406.1
Escherichia coli strain 1585m1 plasmid p1585m1_B, complete sequence	25481	47157	6%	0	99.96%	113047	CP086388.1
Citrobacter freundii strain Cf.1 chromosome, complete genome	25481	39440	6%	0	99.96%	5153556	CP085642.1
Klebsiella pneumoniae strain QD23 chromosome, complete genome	25475	1.75E+05	9%	0	99.96%	5803733	CP042858.1
Citrobacter freundii strain Cf52 plasmid pCf52, complete sequence	25475	77541	8%	0	99.96%	219342	KY887592.1
Citrobacter freundii strain F3517 plasmid pF3517-1, complete sequence	25475	54392	7%	0	99.96%	139338	CP137177.1
Citrobacter freundii strain Survcare457 plasmid pCF_Surv457-KPC2, complete sequence	25473	44814	6%	0	99.96%	83074	CP104961.1
Klebsiella pneumoniae strain KP1572 plasmid pIMP1572, complete sequence	25470	65626	7%	0	99.95%	142993	MH464586.1
Enterobacter hormaechei strain E5 plasmid pE5_003, complete sequence	25470	40243	5%	0	99.95%	86404	CP042574.1
Aeromonas hydrophila strain K522 plasmid pK522-MOX, complete sequence	25470	43627	7%	0	99.95%	176681	CP118701.1

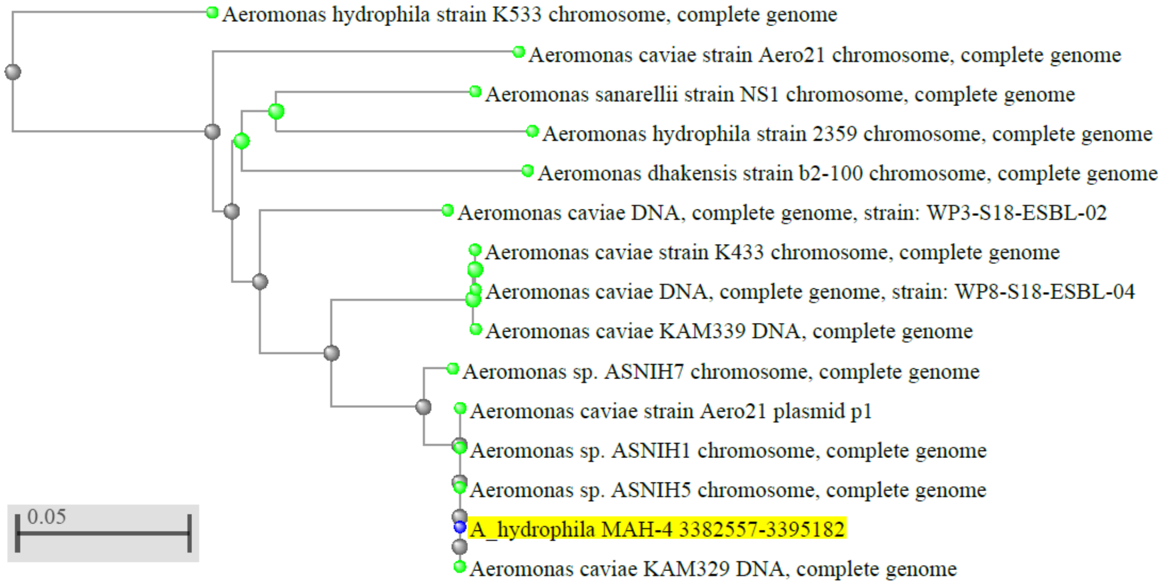

Supplemental Figure S1. Phylogenetic analysis of Trb operon. BLAST Tree was used to compared the chromosomal DNA encoding a Trb operon (trbK-virD4-trbBCDEJKLFGI: 3,382,557-3,395,182) from *Aeromonas hydrophila* MAH-4 against members of the Aeromonadaceae/Succinivibrionaceae group (taxid:135,624).

Table S3. Mobile genetic elements on *Aeromonas hydrophila* pMAH-1

**MGE**	**MGE family**	**Location**	**Origin**	**Acc.#**	**Score (bits)**	**E-value**
ISPst3	IS21	325,934 - 992	*Pseudomonas stutzeri*	AB088753	4726	0.0
ISPa60	ISAs1	1,072 – 2,280	*Pseudomonas aeruginosa*		2381	0.0
ISAhy2	IS630	2,543 – 3,700	*Aeromonas hydrophila*	FM877486	2256	0.0
IS6100	IS6	30,878 – 31,703	*Mycobacterium fortuitum*	X53635	1637	0.0
IS26	IS6	34,991 – 35,810	*Proteus vulgaris*	X00011	1624	0.0
ISCfr1	IS1182	39,539 – 41,155	*Citrobacter freundii*	AF550415	3205	0.0
IS6100	IS6	52,786 – 53,665	*Mycobacterium fortuitum*	X53635	1744	0.0
TnAs1	Tn3	42,984 - 45,884	*Aeromonas salmoncida*	CP022426	5618	0.0
Tn6082	Tn3	15,120 – 18,005	*Erwinia amylovora*	M96392	5642	0.0
**Composite transposons**						
IS6100 - flanked		30,877 - 53,665				
